# Sakrale Fragilitätsfrakturen: Risikofaktoren und Ergebnisse nach Zementsakroplastie

**DOI:** 10.1007/s00132-022-04323-9

**Published:** 2022-11-09

**Authors:** Julian Ramin Andresen, Sebastian Radmer, Axel Prokop, Guido Schröder, Hans-Christof Schober, Reimer Andresen

**Affiliations:** 1 Medizinische Fakultät, Sigmund Freud Privatuniversität, Freudplatz 3, 1020 Wien, Österreich; 2 Zentrum für Bewegungsheilkunde, Facharztpraxis für Orthopädie, Berlin, Deutschland; 3 Klinik für Unfallchirurgie, Sindelfingen, Klinikverbund Südwest, Akademisches Lehrkrankenhaus der Universität Tübingen, Tübingen, Deutschland; 4 Abteilung für Orthopädie und Unfallchirurgie, Warnow Klinik Bützow, Bützow, Deutschland; 5 Klinik für Innere Medizin IV, Klinikum Südstadt Rostock, Akademisches Lehrkrankenhaus der Universität Rostock, Rostock, Deutschland; 6 Institut für Diagnostische und Interventionelle Radiologie/Neuroradiologie, Westküstenklinikum Heide, Akademisches Lehrkrankenhaus der Universitäten Kiel, Lübeck und Hamburg, Heide, Heide, Deutschland

**Keywords:** Knochenmineralgehalt, Osteoporose, Retrospektive Studie, Sakrum, Vitamin-D-Mangel, Bone mineral density, Osteoporosis, Retrospective Study, Os sacrum, Vitamin D deficiency

## Abstract

**Hintergrund:**

Ziel der Untersuchung bei PatientInnen mit Fragilitätsfrakturen des Os sacrum (FFS) war die Erfassung von vorhandenen Risikofaktoren sowie der klinischen Ergebnisse nach Zementsakroplastie (ZSP).

**PatientInnen und Methoden:**

Retrospektiv wurden 68 PatientInnen (64 Frauen, 4 Männer) mit stattgehabten FFS nachuntersucht. Anhand von CT- und MRT-Schnittbildern erfolgte eine Fraktureinteilung nach Denis et al. sowie Rommens und Hofmann. Bei allen PatientInnen wurde eine Knochenmineralgehaltsbestimmung mittels QCT durchgeführt. Unter Berücksichtigung von Anamnese und Röntgenaufnahmen wurden Begleiterkrankungen sowie zentrale und periphere Frakturen miterfasst. Vitamin-D-Werte wurden zusätzlich bestimmt. Nach einem frustranen konservativen Therapieversuch erfolgte eine ZSP. Anhand der Schmerzentwicklung, der körperlichen Selbstständigkeit, der PatientInnen-Zufriedenheit, der Komplikationsrate und der Mortalität wurden die Ergebnisse dokumentiert.

**Ergebnisse:**

Das Alter der Frauen betrugt Ø 83,2 (72–99), dass der Männer Ø 77,8 (76–85) Jahre. Zu 42,4 % fand sich eine Denis-Typ-1-, zu 4,2 % eine Denis-Typ-2-, zu 0 % eine Denis-Typ-3-, zu 43,3 % eine Denis-Typ-1–2- und zu 10,1 % eine Denis-Typ-1–2–3-Frakturzone. Es fand sich ein FFP-Typ-II a-bis -II c-Frakturgeschehen zu 88,2 %, ein FFP-Typ III c zu 7,4 % sowie ein FFP-Typ IV b zu 4,4 %. Bei 68,8 % fanden sich bilaterale FFS. Der Knochenmineralgehalt (KMG) betrug im Ø 35,4 (2–74) mg/ml, der Vitamin-D-Wert im Ø 8,8 (0–28) nmol/l. Weitere osteoporoseassoziierte Frakturen fanden sich in circa 50 %. Nach der ZSP zeigten die PatientInnen eine schnelle und signifikante (*p* < 0,001) Schmerzreduktion sowie nachhaltige klinische Verbesserung.

**Schlussfolgerung:**

Als Frakturrisikofaktoren von FFS fanden sich das weibliche Geschlecht, das hohe Alter, eine vorhandene Osteoporose und ein schwerer Vitamin-D-Mangel. PatientInnen mit nichtdislozierten FFS, welche schmerzbedingt nicht zu mobilisieren waren, profitierten von einer ZSP nachhaltig.

## Abstract (en)

**Background:**

The objective of the present study on patients with fragility fractures of the sacrum (FFS) was to assess existing risk factors and clinical outcomes after cement sacroplasty (CSP).

**Patients and methods:**

68 patients (64 women, 4 men) with previous FFS were followed up retrospectively. CT and MRI images were used to classify fractures according to Denis et al. and Rommens and Hofmann. Bone mineral content was determined by QCT in all patients. Concomitant diseases as well as central and peripheral fractures were recorded, considering the patient’s medical history and X‑ray images. Vitamin D levels were also determined. If conservative therapy was unsuccessful, CSP was performed. The results were documented on the basis of pain development, physical independence, patient satisfaction, complication rate and mortality.

**Results:**

The age of the women in the study was Ø 83.2 (72–99) and that of the men Ø 77.8 (76–85) years. 42.4% had a Denis type 1, 4.2% a Denis type 2, 0% a Denis type 3, 43.3% a Denis type 1–2 and 10.1% a Denis type 1–2–3 fracture zone. FFP type II a to II c fractures were found in 88.2%, FFP type III c in 7.4% and FFP type IV b in 4.4%. Bilateral FFS were found in 68.8%. The average bone mineral content (BMC) was 35.4 (2–74) mg/ml, and the average vitamin D value was 8.8 (0–28) nmol/l. Other osteoporosis-associated fractures were found in around 50% of the patients. After CSP, patients showed a rapid and significant (*p* < 0.001) reduction in pain and sustained clinical improvement.

**Conclusion:**

FFS fracture risk factors were found to be female gender, advanced age, existing osteoporosis and severe vitamin‑D deficiency. Patients with non-displaced FFS who could not be mobilised due to pain experienced sustained benefit from CSP.

Insuffizienzfrakturen des Beckens, einschließlich des Sakrums, stellen immer häufiger bei älteren PatientInnen mit reduzierter Knochenqualität eine Herausforderung im klinischen Alltag dar. Bedingt durch Mikrobewegungen in den Frakturzonen stehen immobilisierende Schmerzen im Vordergrund, welche nicht selten eine zeitnahe Mobilisierung verhindern und damit die konservative Therapie an ihre Grenzen führt. Für die Schaffung einer Primärstabilität gewinnt die Zementaugmentation bei nicht dislozierten Frakturen eine zunehmende Bedeutung hinsichtlich Schmerzreduktion und damit möglich werdenden Mobilisierung.

Fragilitätsfrakturen des Os sacrum (FFS), Synonym: Insuffizienzfrakturen des Sakrums, allein oder in Kombination mit „fragility fractures of the pelvis“ (FFP) werden in letzter Zeit immer häufiger detektiert, wobei aufgrund der steigenden Lebenserwartung die Inzidenz weiter zunehmen wird [23, 42]. Für die FFS wird eine Inzidenz von ca. 2–5 % vermutet, bei Patientinnen > 80 Jahre noch deutlich höher [18, 44], wobei genaue Zahlen nicht vorliegen. Als wichtigste Risikofaktoren gelten das weibliche Geschlecht [24], das Alter > 70 Jahre und eine vorhandene Osteoporose [8, 48]. Für FFP konnte gezeigt werden, dass bei ca. 80 % der Patientinnen zusätzlich ein Vitamin-D-Mangel vorlag [11, 32]. Aufgrund einer Veränderung der Wirbelsäulenbiomechanik nach lumbosakraler Fusion stellt die FFS eine zunehmende Komplikation dar [21]. Eine Radiochemotherapie bei Tumoren im kleinen Becken ist dosisabhängig ein weiterer unabhängiger Risikofaktor für das Auftreten von FFS [33].

Die Standardtherapie der FFS ist bisher eine konservative Behandlung mit Bettruhe und adjuvanter medikamentöser Schmerztherapie, gefolgt von Mobilisierung im Gehwagen oder an Unterarmgehstützen mit schmerzadaptierter Belastung [9]. Problematisch bei der konservativen Therapie ist das erhöhte Risiko von Komplikationen, wie tiefen Venenthrombosen, konsekutiven Lungenarterienembolien, Pneumonien, Dekubitalgeschwüren, Depressionen, des Weiteren kommt es durch die Immobilisierung zu einem fortschreitenden Muskel- und Knochenabbau [9, 29]. Die Ausbildung einer Pseudarthrose mit persistierenden Beschwerden ist ein weiteres Problem des konservativen Vorgehens [29]. Bei Patientinnen mit starken, invalidisierenden Schmerzen ist die Mortalitätsrate unter der konservativen Therapie inakzeptabel hoch [4].

Als alternative minimal-invasive Behandlungsform bietet sich die Einbringung von Zement über Hohlnadeln analog der Vertebroplastie an, diese Technik wurde mit gutem Erfolg erstmals von Garant 2002 durchgeführt [17]. Eine rasche und weitestgehende Schmerzreduktion konnte mit diesem Verfahren mit zunehmender Erfahrung nachgewiesen werden [20], wobei als Komplikation nicht immer symptomlose Leckagen vorkommen können [10].

Als chirurgische Behandlungsoption steht die Osteosynthese mit unterschiedlichen Techniken zur Verfügung [36, 43], als häufigste Methode kommt die perkutane, transiliakale Verschraubung zum Einsatz [36, 43, 47].

Ziel der retrospektiven Untersuchung bei PatientInnen mit FFS war die Erfassung von vorhandenen Risikofaktoren unter besonderer Berücksichtigung eines möglichen Vitamin-D-Mangels und einer vorhandenen Osteoporose sowie des klinischen Benefits nach ZSP.

## PatientInnen und Methoden

Die PatientInnen wurden aus vier Zentren (Nr. 2, 3, 5 und 6 der Autorenadressen) rekrutiert und interventionell mittels ZSP im Zentrum 6 behandelt.

Ausgeschlossen wurden PatientInnen mit Verläufen nach einem Hochenergietrauma sowie mit tumorbedingten ossären Destruktionen oder pathologischen Frakturen. Retrospektiv wurden 68 PatientInnen (64 Frauen, 4 Männer) mit stattgehabten FFS nachuntersucht.

Es erfolgte eine Einteilung der Frakturen nach Denis et al. [14] und nach der Klassifikation der „fragility fractures of the pelvis“ (FFP) nach Rommens und Hofmann [40] anhand von CT- (axiale Schichtdicke von 2 mm durchs Becken mit einer auf das Sakrum koronar angulierten reformierten Schichtdicke von 1 und 2 mm, jeweils dokumentiert im Knochen- und Weichteilfenster) und MRT-Schnittbildern (axiale und sagittale T1- und T2-gewichtete 4‑mm-Schnittbilder durchs Becken sowie auf das Sakrum koronar angulierte STIR-Sequenz mit einer Schichtdicke von 2,8 mm).

Bei allen PatientInnen wurden eine Osteodensitometrie mittels QCT (GE Revolution EVO/64 Zeilen CT, Wauwatosa, WI, USA, sowie Mindways Software 3D Volumetric QCT Spine, Austin, Tx, USA) im LWS-Bereich durchgeführt.

Unter Berücksichtigung von Anamnese und Röntgenaufnahmen wurden Begleiterkrankungen sowie zentrale und periphere Frakturen miterfasst. Der Vitamin-D-Spiegel wurde zu den üblichen Laborwerten zusätzlich mitbestimmt. Ein eventuell vorhandener Vitamin-D-Mangel wurde unmittelbar ausgeglichen und entsprechend der DVO-Leitlinie [45] als Dauermedikation fortgesetzt. Eine weiterführende medikamentöse antiosteoporotische, osteoanabole Therapie wurde empfohlen.

Als konservative Maßnahmen durchliefen die PatientInnen zunächst in Abhängigkeit von der Schmerzintensität eine Bettruhe, eine adjuvante medikamentöse Schmerztherapie nach dem WHO-Schema [27] sowie eine Mobilisierung mithilfe eines Gehwagens oder an Unterarmgehstützen mit schmerzadaptierter Belastung. Nach einem frustranen 3‑wöchigen Verlauf mit weiterhin bestehenden immobilisierenden Schmerzen > 5 auf der VAS erfolgte nach einer interdisziplinären Fallkonferenz die Zuweisung zur ZSP [5]. Die Zementaugmentation erfolgte CT-gesteuert mit einem Low-Dose-Programm. Mögliche Zementleckagen wurden mittels CT-Schnittbildgebung (kraniokaudale Spirale mit einer Schichtdicke von 0,625 mm und einer 2 mm axialen, 1 mm angulierten koronaren und 1 mm sagittalen Reformation) am ersten postoperativen Tag detektiert. Jeder Zement außerhalb der kortikalen Begrenzung des Os sacrum, einschließlich der Neuroforamina, wurde als Leckage gewertet.

In Abhängigkeit der Klinik wurden die PatientInnen dann nach 4–6 Tagen in die frührehabilitative Komplextherapie verlegt oder nach Hause in die ambulante Weiterbehandlung entlassen.

Im weiteren Verlauf wurden dann über 24 Monaten die Schmerzentwicklung mittels VAS, die Selbstständigkeit mittels einem modifizierten Hamburger-Barthel-Index (HBI, Tab. 1; [30]), die Komplikationen einschließlich Tod und die PatientInnenzufriedenheit dokumentiert.

**Table 1:** 

		Items
Essen	Selbstständig, unabhängig	10
	Benötigt etwas Hilfe	5
	Nicht selbstständig	0
Bett/Rollstuhltransfer	Unabhängig in allen Phasen	15
	Geringe Hilfen oder Beaufsichtigung	10
	Erhebliche Hilfe beim Transfer und Lagewechsel	5
	Nicht selbstständig	0
Waschen	Unabhängig in allen Phasen der Tätigkeit	5
	Nicht selbstständig	0
Toilettenbenutzung	Unabhängig in allen Phasen	10
	Benötigt Hilfe	5
	Nicht selbstständig	0
Baden	Unabhängig bei Voll- oder Duschbad	5
	Nicht selbstständig	0
Gehen auf Flurebene bzw. Rollstuhl fahren	Unabhängig beim Gehen über 50 m	15
	Kann mit Hilfsmitteln 50 m gehen	10
	Nicht selbstständig, mit Rollstuhl sind 50 m möglich	5
	Nicht selbstständig beim Gehen oder Rollstuhl fahren	0
Treppensteigen	Unabhängig bei der Bewältigung einer Treppe	10
	Benötigt Hilfe	5
	Nicht selbstständig, auch nicht mit Hilfe	0
An- und Auskleiden	Unabhängig	10
	Benötigt Hilfe	5
	Nicht selbstständig, auch wenn Hilfe gewährt wird	0
Stuhlkontrolle	Ständig kontinent	10
	Gelegentlich inkontinent	5
	Häufiger/ständig inkontinent	0
Urinkontrolle	Ständig kontinent	10
	Gelegentlich inkontinent	5
	Häufiger/ständig inkontinent	0
*Summe (Bereich 0–100)*		

## Statistik

Die statistische Analyse der Ergebnisse wurde mit der Prism 8 Software (GraphPad Software, Inc., San Diego, CA, USA) durchgeführt. Der Students-t-Test wurde zum Mittelwertvergleich zwischen zwei Gruppen (KMG- oder Vitamin-D-Werte zwischen PatientInnen mit einer unilateralen und bilateralen Fraktur) herangezogen. Gleichzeitig wurden die Effektstärken nach Cohen berechnet und Werte < 0,5 als kleiner, zwischen 0,5 und 0,8 als mittlerer sowie > 0,8 als großer Effekt angenommen. Die statistische Signifikanz wurde mit signifikant = *p* < 0,05, hoch signifikant = *p* < 0,005 und sehr hoch signifikant = *p* < 0,0005 gekennzeichnet.

## Ethik

Die nachfolgende retrospektive, multizentrische, klinische Untersuchung wurde durch die zuständige regionale Ethikkommission der Universitätsmedizin Rostock geprüft und genehmigt (Nr. A 2020-0015).

## Ergebnisse

### PatientInnen

Das Alter der Frauen betrugt Ø 83,2 (72–99), dass der Männer Ø 77,8 (76–85) Jahre.

### Frakturtypen und -häufigkeiten

**Figure 1:**
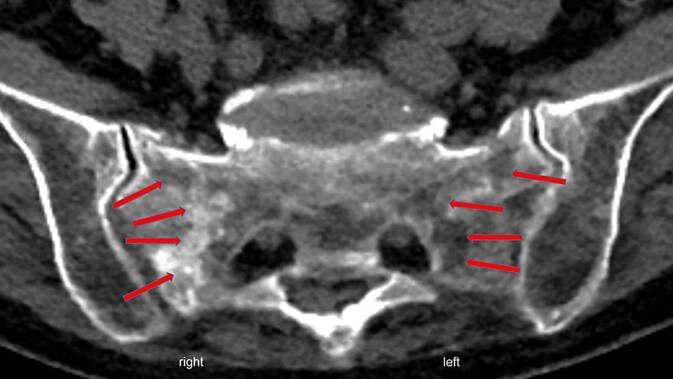

Zu 43,7 % fand sich eine Denis-Typ-1-, zu 4,2 % eine Denis-Typ-2-, zu 0 % eine Denis-Typ-3-, zu 43 % eine Denis-Typ-1–2- und zu 9,1 % eine Denis-Typ-1–2–3-Frakturzone. Es fand sich ein FFP-Typ-II a- bis -II c-Frakturgeschehen zu 88,2 %, ein FFP-Typ-III c zu 7,4 % sowie ein FFP-Typ-IV b zu 4,4 %. Mit 68,8 % fanden sich bilaterale FFS, somit lagen 115 einzelne FFS bei 68 PatientInnen vor. Als Hinweis für ein unterschiedliches Alter der FFS fanden sich bei den bilateralen Frakturen meist unterschiedlich stark ausgeprägte Ödeme und zum Teil seitendifferente Sklerosierungen im Bereich der Frakturzonen in der CT- (Abb. 1) und MRT-Bildgebung.

### Knochenmineralgehalt und Vitamin D

**Figure 2:**
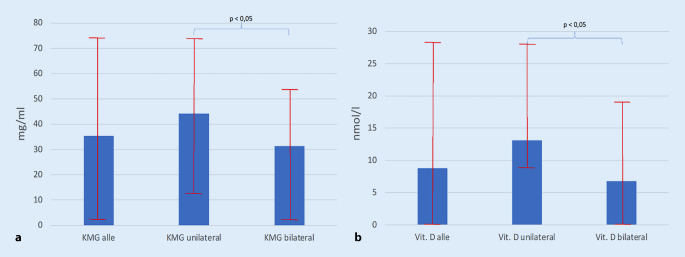

Der KMG der LWS betrug im Ø 35,4 (2–74) mg/ml für alle PatientInnen, bei den unilateralen Frakturen Ø 44,3 (12–74) mg/ml und bei den bilateralen Frakturen Ø 31,3 (2–54) mg/ml (Abb. 2a). Der Vitamin-D-Wert lag für alle PatientInnen bei Ø 8,8 (0–28) nmol/l ≙ Ø 4,93 (0–11,2) ng/ml, bei den unilateralen Frakturen bei Ø 13,1 (8–28) nmol/l ≙ Ø 5,24 (3,2–11,2) ng/ml und bei den bilateralen Frakturen bei Ø 6,8 (0–18) nmol/l ≙ Ø 2,72 (0–7,2) ng/ml, (Abb. 2b). Die Unterschiede zwischen den unilateralen und bilateralen Frakturen sind für die Knochenmineralgehalts- und Vitamin-D-Werte signifikant (*p* < 0,05).

### Zementsakroplastie

Nach einem konservativen Behandlungsversuch lagen die Schmerzen der zur ZSP zugewiesenen, nicht zu mobilisierenden PatientInnen bei 8,7 ± 0,59 Scorepunkten auf der VAS.

**Figure 3:**
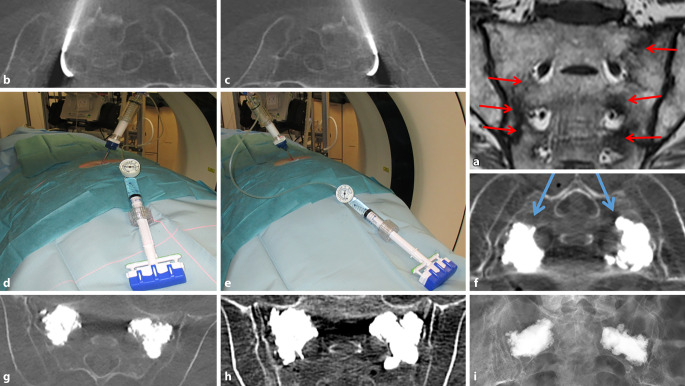

**Figure 4:**
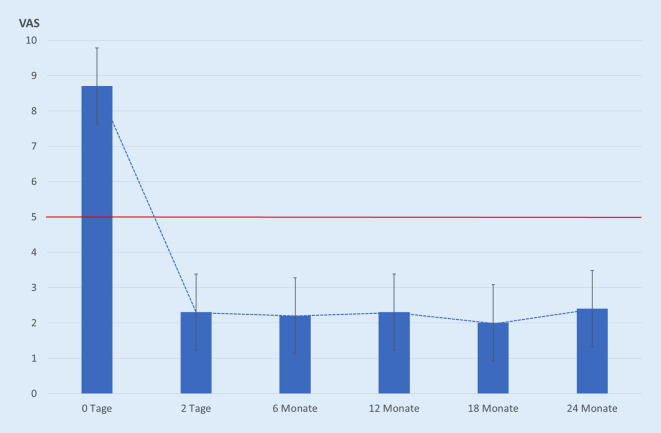

**Figure 5:**
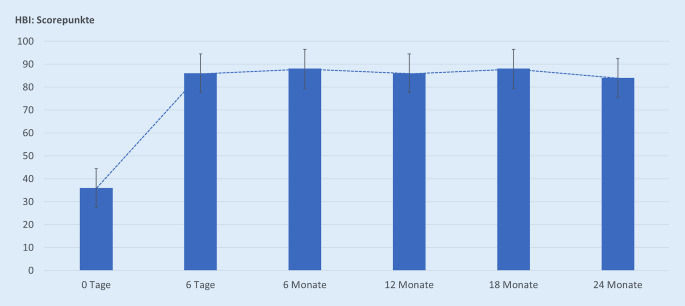

Bei allen PatientInnen ließ sich die ZSP technisch gut durchführen. Bei Vorliegen einer bilateralen Fraktur wurden diese in einer Sitzung versorgt. Pro Fraktur wurden im Ø 6 (3–10) ml PMMA eingebracht. Eine Zementleckage fand sich bei 6 von 68 (8,8 %) PatientInnen, keine der Leckagen war symptomatisch. Ein Beispiel für eine ZSP ist in Abb. 3 illustriert. Zwei Tage nach Zementaugmentation zeigten die PatientInnen eine schnelle und signifikante (*p* < 0,001, Effektstärke > 0,8) Schmerzreduktion (Abb. 4), welche rasch eine Mobilisierung ermöglichte und zu einer nachhaltigen klinischen Verbesserung führte (Abb. 5). Die PatientInnenzufriedenheit war nach der ZSP durchweg gut (Tab. 2).

**Table 2:** 

Schmerzrückgang	Schnell, gut und nachhaltig
Entwicklung der Selbstständigkeit	Deutlich und nachhaltig
Subjektive Zufriedenheit	Sehr gut nach der ZSP, gut im Verlauf

### Komplikationen und Mortalität

Während des stationären Aufenthaltes kam es zu keinem Todesfall. Eine postinterventionell bedingte Blutung oder Infektion konnte für alle PatientInnen ausgeschlossen werden. Durch einen Sturz in häuslicher Umgebung erlitten 2 PatientInnen eine Schenkelhalsfraktur, 4 PatientInnen entwickelten eine zusätzliche Insuffizienzfraktur am Achsenskelett, eine erneute Beckenfraktur konnte nach klinischer Untersuchung im Verlauf von 24 Monaten verneint werden.

Zur Abschätzung der Selbstständigkeit erreichten die PatientInnen nach ZSP 83 ± 6 Scorepunkte am Ende von 24 Monaten auf dem HBI (Abb. 5). Es erreichten jedoch nur 23 von 68 (33,8 %) der behandelten PatientInnen die körperliche Fitness wie vor dem Frakturereignis. Die Mortalität betrug nach 12 Monaten 8,4 %, nach 18 Monaten 14,2 % und nach 24 Monaten 20,5 %. Ursächlich standen hier eine Pneumonie, tiefe Beinvenenthrombose mit konsekutiver Lungenarterienembolie, Urosepsis, Herzinsuffizienz/Herzinfarkt, Apoplex und Tumorleiden im Vordergrund.

### Zusätzliches Krankheitsprofil

Bei 30 von 68 (44,1 %) aller PatientInnen fand sich zu mindestens eine stattgehabte Sinterungsfraktur im Bereich der BWS und LWS. Weitere osteoporoseassoziierte Frakturen wie distale Radius-, proximale Humerus‑, Schenkelhals‑, Rippen- und Sternumfrakturen fanden sich anamnestisch bei 33 von 68 (48,5 %) allen PatientInnen. Eine Hypokalzämie fand sich bei 35 % und ein sekundärer Hyperparathyreoidismus bei 48 % aller PatientInnen. Eine zusätzliche Lungenerkrankung fand sich bei 23,6 %, eine kardiovaskuläre Erkrankung bei 45,2 %, eine Hypertonie bei 77,1 %, eine Niereninsuffizienz bei 34,2 %, ein Diabetes mellitus Typ II bei 72,8 %, eine pAVK bei 70,3 % und eine Adipositas mit einem BMI von > 30 kg/m^2^ bei 60,2 % aller PatientInnen. Ein unterschiedlich ausgeprägter Nikotinkonsum wurde von 48,3 % aller PatientInnen angegeben.

## Diskussion

Wie in anderen Arbeiten bestätigen sich in unserem PatientInnenkollektiv mit FFS als Risikofaktoren das fortgeschrittene Alter [23, 24, 42, 49], das weibliche Geschlecht [18, 24], ein schwerer Vitamin-D-Mangel [11, 32] und eine Osteoporose [4, 32], welches sich ausgeprägt auch im Os sacrum findet [49]. Andererseits ist eine FFS ein starker Indikator für das Vorliegen einer manifesten Osteoporose [41].

Die KMG-Werte der QCT-Messung am Achsenskelett lagen deutlich unterhalb der Schwelle zur Osteoporose von 80 mg/ml [15] und unterhalb der Schwelle von 60 mg/ml, wo das Frakturrisiko am Achsenskelett deutlich ansteigt [2], wobei sich mit durchschnittlich 31,3 mg/ml signifikant die niedrigsten Werte bei bilateralen FFS fanden. Die zusätzlichen Sinterungsfrakturen bei 44,1 % aller PatientInnen am Achsenskelett und Frakturen bei 48,5 % aller PatientInnen im peripheren Skelettbereich untermauern das Vorliegen einer schweren, klinisch manifesten Osteoporose in unserem PatientInnenkollektiv. Das Ausmaß des niedrigen KMG und des Vitamin-D-Mangels korrelierte mit der Schwere der Frakturmorphologie im Os sacrum (Abb. 2a, b) Die Frakturentwicklung stellt hierbei einen dynamischen Prozess dar, wobei bilaterale FFS eine zunehmende Instabilität entwickeln können [34], welches dann bei zunehmender Dislokation kurzfristig eine Osteosynthese notwendig macht [22, 36, 40].

Die Anzahl und prozentuale Verteilung von zusätzlichen Begleiterkrankungen werden in ähnlicher Weise auch von Maier et al. [32] gefunden.

Bei nicht dislozierten FFS lässt sich mittels ZSP durch die Einbringung einer PMMA-Zementplombe in die entsprechende Frakturzone eine lokal erhöhte Stabilisierung [1] und damit Minimierung von Mikrobewegungen erreichen, welches zu einer signifikanten Schmerzreduktion führt [5, 7, 20, 46]. Auch bei unseren PatientInnen kam es zu einer schnellen und signifikanten Schmerzreduktion (Abb. 4) mit einer schnell eintretenden, deutlichen Verbesserung der Selbstständigkeit (Abb. 5) und guten PatientInnenzufriedenheit (Tab. 2). Die schnelle, signifikante und nachhaltige Schmerzreduktion ist der größte Nutzen für die PatientInnen nach einer ZSP. Von vielen Arbeitsgruppen wurde dieses gefunden [5–7, 17, 20, 28, 46] und durch vergleichbare Ergebnisse in Multicenterstudien [16, 26], systematischen Reviews sowie Metaanalysen untermauert [7, 12, 13, 31, 48].

Die Mortalitätsraten liegen für unsere PatientInnen nach einer ZSP mit 8,4 % nach 12 Monaten deutlich niedriger im Vergleich zu PatientInnengruppen nach einer konservativen Therapie, wobei Raten von 17,5–23,5 % beschrieben werden [4, 32, 38], genaue Vergleichswerte zwischen konservativer und interventioneller Therapie liegen für einen Zeitraum von 18 bzw. 24 Monaten nicht vor.

Zur Minimierung von PMMA-Zementleckagen [10] sind eine genaue Kenntnis der Sakrumanatomie, der Frakturmorphologie und möglichen Instabilität [14, 40], eine gute Bildgebung bei der Zementeinbringung [39], eine exakte Planung der möglichen Zugangswege [3, 35], ein Wissen über eine optimal einzubringende Zementmenge sowie dem Verhalten von möglichst hochviskösen Zementen [5, 35] und Erfahrung im Umgang mit osteoplastischen Verfahren, wozu die ZSP gehört (Abb. 3; [5]), zwingend nötig. Im Gegensatz zur konventionellen Vertebroplastietechnik [10] ist die ZSP [5] zur Vermeidung von Leckagen das sichere Verfahren.

Nach der Intervention ist es weiter notwendig, die vorhandene Osteoporose zu behandeln und eine Frakturheilung medikamentös zu unterstützen, wobei eine osteoanabole Medikation [37, 50] gewählt werden sollte. Dieses beinhaltet auch einen entsprechenden Ausgleich des schweren Vitamin-D-Mangels [19]. Eine zusätzliche Physiotherapie kann einer weiteren Verschlechterung des muskuloskelettalen Systems entgegenwirken [25].

## Limitationen

Bei der vorliegenden Studie handelt es sich um eine retrospektive Untersuchung. Es gibt keine direkte konservative oder chirurgische Vergleichsgruppe zur durchgeführten ZSP-Gruppe.

## Fazit für die Praxis

- Eine Vitamin-D-Substitution und antiosteoporotische Medikation sind notwendig, unabhängig ob eine konservative, interventionelle oder osteosynthetische Therapie der FFS (Fragilitätsfrakturen des Sakrums) erfolgt.
- Vorzugsweise sollte zur beschleunigten Frakturheilung eine osteoanabole Medikation gewählt werden.
- PatientInnen mit nicht dislozierten FFS und frustranem konservativen Therapieversuch profitieren von einer anschließenden Zementsakroplastie schnell und nachhaltig.

## Abkürzungen

BMI: Body Mass Index
BWS: Brustwirbelsäule
CT: Computertomografie
DVO: Dachverband Osteologie
FFP: Fragilitätsfrakturen des Beckens
FFS: Fragilitätsfrakturen des Sakrums
GE: General Electric
HBI: Hamburger-Barthel-Index
KMG: Knochenmineralgehalt
LWS: Lendenwirbelsäule
MRT: Magnetresonanztomografie
PAVK: Periphere arterielle Verschlusskrankheit
PMMA: Polymethylmethacrylat
QCT: Quantitative Computertomografie
STIR: Short-Tau-Inversion-Recovery
VAS: Visuelle Analogskala
Vit: Vitamin
ZSP: Zementsakroplastie
Ø: Durchschnitt
